# Cephalopod Ink: Production, Chemistry, Functions and Applications

**DOI:** 10.3390/md12052700

**Published:** 2014-05-12

**Authors:** Charles D. Derby

**Affiliations:** Neuroscience Institute and Department of Biology, Georgia State University, P.O. Box 5030, Atlanta, GA 30302-5030, USA; E-Mail: cderby@gsu.edu; Tel.: +1-404-413-5393

**Keywords:** Cephalopoda, cuttlefish, funnel organ, ink, ink sac, melanin, neuroecology, octopus, predator-prey, squid

## Abstract

One of the most distinctive and defining features of coleoid cephalopods—squid, cuttlefish and octopus—is their inking behavior. Their ink, which is blackened by melanin, but also contains other constituents, has been used by humans in various ways for millennia. This review summarizes our current knowledge of cephalopod ink. Topics include: (1) the production of ink, including the functional organization of the ink sac and funnel organ that produce it; (2) the chemical components of ink, with a focus on the best known of these—melanin and the biochemical pathways involved in its production; (3) the neuroecology of the use of ink in predator-prey interactions by cephalopods in their natural environment; and (4) the use of cephalopod ink by humans, including in the development of drugs for biomedical applications and other chemicals for industrial and other commercial applications. As is hopefully evident from this review, much is known about cephalopod ink and inking, yet more striking is how little we know. Towards closing that gap, future directions in research on cephalopod inking are suggested.

## 1. Introduction

“When the Sepia is frightened and in terror, it produces this blackness and muddiness in the water, as it were a shield held in front of the body.”—Aristotle, *The History of Animals, Book IV* (*ca.* 350 BC). Translated by Arthur Leslie Peck and Edward Seymour Forster. *Aristotle XII: Parts of Animals Movement of Animals, Progression of Animals* (1937).

“I was much interested, on several occasions, by watching the habits of an Octopus or cuttle-fish. Although common in the pools of water left by the retiring tide, these animals were not easily caught. They darted tail first, with the rapidity of an arrow, from one side of the pool to the other, at the same instant discolouring the water with a dark chestnut-brown ink.”—Charles Darwin, *The Voyage of the Beagle* (1839).

“She took a sheet of paper and began to sketch in sepia the head of the hidden man. A work done under the impulse of an emotion has always a stamp of its own.”—Honoré de Balzac, *La Vendetta* (1830).

“To the Italian palate, the harsh, pungent ink is the least desirable part of the squid. As Venetian cooks have shown, it’s only the mellow, velvety, warm-tasting ink of cuttlefish—seppie—that is suitable for pasta sauce, risotto, and other black dishes.”—Marcella Hazan, *Essentials of Classic Italian Cooking* (1992).

Cephalopods have stimulated the scientific minds, artistic emotions and sensory palates of humans for millennia, and as the above quotations reveal, their ink has played a central role in this fascination. Aristotle wrote about inking cuttlefish 2500 years ago. In fact, Aristotle himself has been described by his contemporaries and modern scholars as cephalopodan, with the simile that “he is like the cuttlefish who obscures himself in his own ink when he feels himself about to be grasped” [1].

The goal of this review is to present what is known about cephalopod ink, including its production, functions in the natural environment, including predator-prey interactions, chemical constituents and their role in these functions and its use by humans, including in the development of drugs, therapeutics and commercial and industrial applications.

## 2. Cephalopods: Who Are They and Who Inks

### 2.1. Cephalopod Systematics

**Figure 1:**
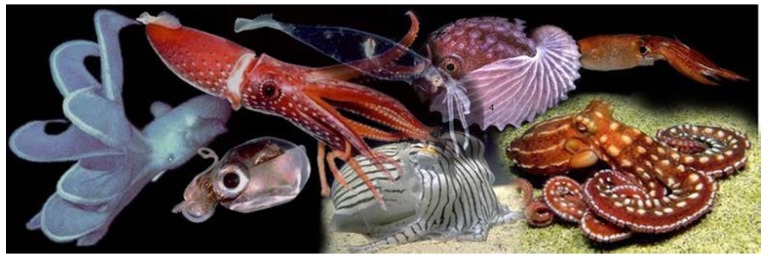
The cephalopods. Representative forms of cephalopods, including *Histioteuthis cerasina*, *Cirroteuthis magna*, *Octopus ornatus*, *Argonauta nodosa*, *Sepioloidea lineolata*, *Iridoteuthis iris*, *Nototodarus hawaiiensis* and *Leachia pacifica*. From Richard E. Young, Michael Vecchione and Katharina M. Mangold [8]. Used with permission from Richard Young and Mark Norman.

Cephalopoda is a class of Mollusca that appeared ~500 million years ago and that lives in the marine environment. Cephalopods are represented by two major extant groups: Nautiloidea (nautilus) and Coleoidea. The coleoids are organized into Octopodiformes (Vampyromorpha, vampire squids; and Octopoda, octopuses) and Decapodiformes (squids and cuttlefishes) (Figure 1). Although cephalopods are represented by relatively few species (~700), they are widely distributed in many different oceanic habitats.

### 2.2. Which Cephalopods Produce Ink?

**Figure 2:**
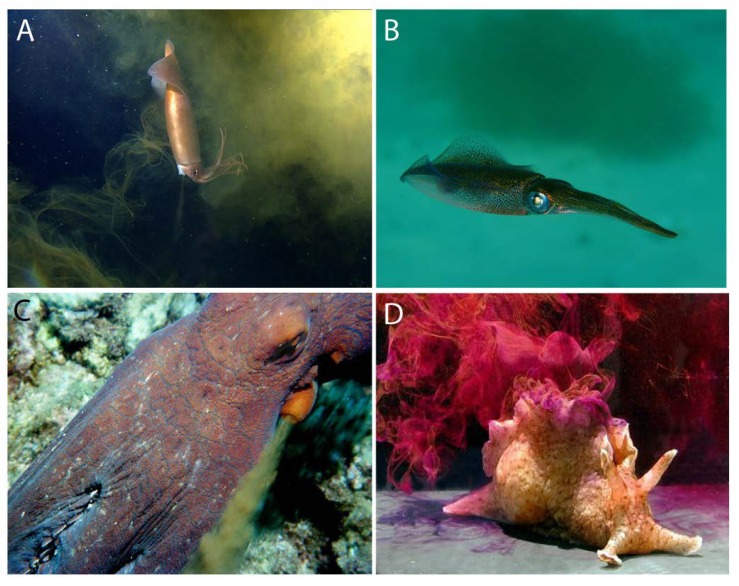
Inking mollusks. Three inking cephalopod mollusks (**A**–**C**) and one inking gastropod mollusk (**D**). (**A**) Humboldt squid, *Dosidicus gigas* (used with permission from the Monterey Bay Aquarium Research Initiative); (**B**) Squid with an ink cloud in the background (used with permission from Klaus Stiefel); (**C**) Octopus escaping and inking (used with permission from Jeffrey N. Jeffords); (**D**) Sea hare, *Aplysia californica* (used with permission from Genevieve Anderson). The sea hare’s use of ink as a chemical defense has been well studied (Section 5) and can be used as a model for exploring the cephalopods’ use of ink as a chemical defense.

All orders of the Coleoidea have ink sacs and produce ink (Figure 2A–C), but the members of the Nautiloidea do not. There are some coleoids that have secondarily lost their ink sac, such as octopod species in the suborder, Cirrata (=Cirrina), that are mostly deep-sea dwelling [2,3,4], but also include shallow-water nocturnal species [5]. Ink is produced and used by many cephalopod species living in low-light or dark conditions, including the deep sea [6]. The ink sac is present at hatching, so even at a small size and young age, cephalopods can produce and release ink [7].

## 3. What Is Cephalopod Ink?

### 3.1. Ink Is a Mixture of Two Glandular Secretions

**Figure 3:**
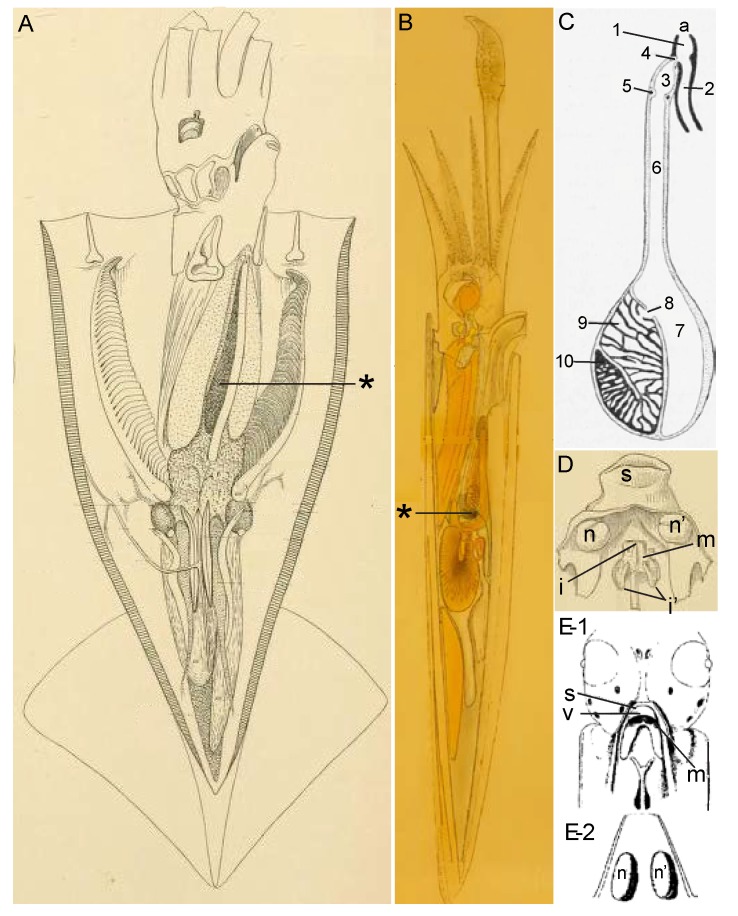
Cephalopods and their ink-producing glands. These 19th century images have been relabeled and retouched for clarity. (**A**,**B**) Squid and its ink sac. (**A**) *Illex illecebrosus*, from Figure 2 of Plate XXXIX of Verrill in 1880 [9]. (**B**) *Doryteuthis pealeii*, from Figure 13 of Williams in 1909 [10]. The ink sac is labeled by an asterisk; (**C**) Ink sac from the cuttlefish, *Sepia officinalis*, from Figure 291 of Lang and Hescheler in 1900 [11]. a, anus; 1, common sac of the rectum and ink sac; 2, rectum; 3, ampulla of the duct of the ink sac; 4, anterior sphincter muscle of the ink sac; 5, posterior sphincter muscle of the ink sac; 6, duct of the ink sac; 7, reservoir of the ink sac; 8, opening of the ink gland; 9, ink gland; 10, basal germinal zone; (**D**,**E**) Funnel organ of squid; (**D**) Ventral view of the funnel organ from *Desmoteuthis tenera* (=*Teuthowenia megalops*) from Figure 2d of Plate LV from Verrill in 1880 [9] and from *Loligo*; (**E-1**) Ventral view and (**E-2**) dorsal view, from Figure 2 of Laurie in 1888 [12]. The funnel organ, located on the funnel near the siphon (s) and siphonal valve (v), is composed of a median organ (m), two lateral cushions (n, n′) and a papilla to each (one medio-dorsal papilla, i, and two lateral papillae, i′).

Cephalopod ink is composed of secretions from two glands. The ink sac with its ink gland produces a black ink containing melanin, and most of what is known about cephalopod ink comes from studying it. A second organ, the funnel organ, is a mucus-producing gland that is much more poorly studied. Figure 3 shows various aspects of these glands, taken from early studies of cephalopods.

#### 3.1.1. Ink Sac

The ink sac has been the object of attention, both casually and scientifically, because of its dramatic black color. Its location is shown in Figure 3A,B. Its basic organization has been long known. In fact, the standard drawings currently used in reviews to depict the anatomy of the ink sac and gland, such as figure 75 of Budelmann *et al.* in 1997 [13], can be traced back to figure 2 of Girod in 1882 [14]. For example, Budelmann *et al.* [13] state that their figure 75 of the ink sac is modified from Mangold *et al.* in 1989 [15] (their figure 107), who state that theirs is modified from Lang and Hescheler in 1900 [11] (their figure 291, reproduced here as Figure 3C), who, in turn, state that theirs is modified from Girod in 1882 [14] (their figure 2). Over the years, there has been much excellent work on the form and function of the ink sac. Besides the early papers from the 19th century cited above and the more recent work on the cell biology and biochemistry of the production of melanin, especially by the Italian scientists, including Nicolaus, Prota, Palumbo, Di Cosmo and many others working on the cuttlefish, *Sepia officinalis* (see Section 4), notable studies on the functional organization of the ink sac include those by Turchini in 1921–1922 [16,17,18], Yung Ko Ching in 1930 [19], Graupner and Fischer in 1934 [20], Ries in 1937 [21], Fioroni in 1962–1963 [22,23,24], Szabo *et al.* in 1963 [25], Vogel and McGregor in 1964 [26], Dill and Herring in 1978 [27] and Wang *et al.* in 2008 [28]. The ink sac, which forms as a diverticulum of the hind gut, consists of several parts (Figure 3C). At the base of the ink sac is the ink gland, which produces the melanin-rich ink, as described in Section 4. The ink gland releases its secretion into the ink sac lumen, where it is stored and eventually released via a duct into the hindgut near the anus. The release of ink from the ink sac is controlled by its muscular walls and a pair of sphincters.

#### 3.1.2. Funnel Organ

The funnel organ is the second gland contributing to the ink secretion, though much less is known about it than the ink sac. Verrill in 1880 [9] provided illustrations of the funnel organ in several squid species (Figure 3D) and without giving the organ a name, described it in the following way: “towards the base (of the siphon), there are two erect, rounded, ear-like flaps, each with a small papilla (*i′*), and a rounded, valve-like, raised median fold and a central papilla (*i*) in front of them.” Hoyle in 1886 [29], in presenting the results of studies of cephalopods from the *Challenger* expedition, named this structure the “organ of Verrill”. Laurie in 1888 [12] provided a histological description of this organ in squid, which allowed him to be the first to correctly identify it as a mucus gland due to its “columnar goblet cells almost entirely filled with a clear transparent substance which stains very darkly with hematoxylin” and with nuclei “surrounded by a small quantity of glandular protoplasm.” Laurie also noted that the surface of the organ was often coated with “large quantities of mucus-like substance which has apparently been excreted from the cells.” In the same year as Laurie’s publication, Weiss in 1888 [30] noted this organ in cuttlefish. However, in fact, the funnel organ was first described in various cephalopods by Müller (1853) [31] nearly three decades earlier. As tactfully put by Williams in 1909 [10], “this description seems, as Brock (1888) has pointed out, to have been overlooked by Verrill, Hoyle, and Laurie.” Ever since then, this structure has been more commonly called the funnel organ rather than the organ of Verrill. More recent studies of relevance to the morphology of the funnel organ include Voss in 1963 [32] and Hu *et al.* in 2010 [33].

#### 3.1.3. Combining the Two Glandular Secretions

Despite the early recognition that the funnel organ is a mucus-producing gland, its function in inking was not realized for some time. In fact, the evidence for its involvement in inking is based more on the process of elimination than any experimental demonstration. First, ink has a significant volume of mucus, usually much more than is present in the ink sac. Second, this mucus must come from somewhere in the area of the funnel, siphon and mantle cavity. Third, the funnel organ, due to its size and location, appears to be the only mucus gland that could secrete this volume of mucus and co-release it with the ink sac’s secretion.

Several authors have stated that the combined secretions of the ink sac and funnel organ, produced in different amounts, leads to ink of different forms, ranging from a diffuse cloud to a discrete object with the general appearance of a squid, and, thus, called a pseudomorph, to longer and thinner forms, called ropes [6,7,8,34]. It is interesting to speculate that these different forms might be produced depending on the type of predatory attack. The work of Bush and Robison [6] comes closest to addressing this. They showed that members of a given species produced different forms of ink, though for each species, one form was more commonly produced than others. Additionally, they showed that each ink form was associated with particular behaviors. For example, individuals releasing pseudomorphs were more likely to produce escape behaviors, while individuals releasing clouds and ropes were more likely to swim next to the ink, presumably favoring crypsis over active escape.

Some deep-water cephalopods produce luminous secretions. According to Bush and Robison [6], species of the sepiolid genus, *Heteroteuthis*, are the only cephalopods known to release luminous secretions from ink sacs [27,35]. Other deep-water cephalopods, such as *Vampyroteuthis infernalis*, release a viscous fluid containing microscopic luminescent particles from their arm tips; in fact, this species does not have an ink sac [6,36]. The release of secretions from both the ink sac and funnel organ is under neural control. There is evidence that the two organs are controlled by separate neural pathways, but this should be viewed with caution, since results are from few species and are based on classical anatomical track-tracing techniques.

The ink sac, including its duct and the anterior and posterior sphincter muscles, is innervated by visceral nerves from the latero-ventral palliovisceral lobe of the posterior subesophageal mass [37,38,39,40,41]. In support of this is Boycott’s [42] demonstration that electrical stimulation of the palliovisceral lobe causes ink release. This innervation appears to be glutamatergic [43], which may involve NMDA-type receptors on muscle in the ink sac duct [38,44].

The innervation of the funnel organ is less well characterized. Young [45] described the funnel organ of *Loligo* as being innervated by a branch of the posterior funnel nerve, which originates in the antero-ventral palliovisceral lobe of the posterior subesophageal mass. This nerve branch, which contains relatively large-diameter (25 μM) fibers, innervates muscles at the base of the glands and may also innervate the gland cells themselves [45]. Other branches of the posterior funnel nerve, as well as anterior and median funnel nerves innervate the funnel [45], but it was not reported if these branches innervate the funnel organ itself. Somewhat different from Young’s [45] description for *Loligo* is Shigeno and Yamamoto’s [40] description of the funnel organ in the pygmy cuttlefish, *Idiosepius paradoxus*. The funnel organ of this species appears to be innervated by the median and/or anterior funnel nerves and, thus, would be connected to the posterior pedal lobe of the middle subesophageal mass and ventral magnocellular lobe of the periesophageal mass. In octopus, anterior and posterior funnel nerves, originating in the middle and posterior subesophageal masses, respectively, have been described as innervating the funnel, though they were not specifically described as innervating the funnel organ itself [37,46]. Embryonic and postlarval squid (*Sepioteuthis lessoniana*) also have funnel nerves originating in the posterior subesophageal mass [47].

Thus, it appears that the ink sac and funnel organ may be innervated by different neural pathways and, thus, can be independently controlled, though this is not certain. There remains much to be determined about the innervation of these organs and its significance, including whether the type of ink produced, such as the relative amounts of secretion from these two organs, depends on the threat perceived by the cephalopod.

## 4. Chemical Constituents of Ink

### 4.1. Methodology Matters

To understand the roles played by chemicals in natural environments, it is critical to know the natural rates of release of these chemicals from their biological sources and the fluid dynamics in these natural environments and then to examine the effects of these chemicals in the laboratory or field at these ecologically relevant concentrations and temporal-spatial dynamics. The importance of this approach is highlighted in the works of leading chemical ecologists [48,49,50,51,52].

Along these lines, how ink is collected from ink sacs of cephalopods can affect what chemical structures and concentrations are identified in ink. Madaras *et al.* [53] emphasized this point, based on studies of ink from the squid, *Sepioteuthis australis*. They compared two collection methods. One method (“syringe”) used a syringe to collect ink from the duct end of the ink sac of freshly-killed squid and, thus, tried to simulate natural release with as little manipulation of and damage to the ink sac as possible. The second method (“milking”) used animals dead within 24 h, whose ink sac was then dissected out; the ink sac duct was cut, and the contents of the sac were milked into a tube by running forceps along its length, which optimizes the amount of ink collected, but is more likely to include material from damaged tissue. The “syringe” method identified DOPA (=l-3,4-dihydroxyphenyl-alanine, or l-dopa), dopamine and taurine as constituents of ink, but failed to find either epinephrine (a catecholamine derived from tyrosine, like DOPA) or proteins (neither tyrosinase nor other proteins, including those in the melanin-free fraction or bound to melanin). On the other hand, the “milking” method yielded tyrosinase and epinephrine, which, according to Madaras *et al.*, may be due to disruption of the parts of the ink gland that normally do not release their contents into the ink sac, either due to damage during collection or by autolysis after death of the animal and before collection.

A review of the literature shows that researchers have typically collected ink in four general ways. Each method has its own advantages, depending on the goals of the study. The most ecologically relevant method is collecting ink from sea water after it has been released from live, freely behaving animals [54,55]. This method allows the collection of the combined secretion of the ink sac and the funnel organ, which is the form naturally released. The potential disadvantages of this method are unquantifiable dilution of the ink secretion and contamination of ink with other chemicals in the water. A second method is the syringe technique of Madaras *et al.* [53], in which ink is taken from an ink sac of recently-killed animals without milking or squeezing the ink sac or gland. A third method is similar to the second except that the ink sac of freshly killed animals is squeezed, expressed or milked from freshly-killed animals [56,57,58,59]. The syringe method has the advantage over the milking method by reducing the likelihood of damaging the gland during collection and the consequent collection of chemicals that are not naturally released. A fourth method is homogenizing the ink sacs, which yields a mixture that includes chemicals not only in the ink itself, but also in the tissues of the ink sac and gland and which are not released during inking [60]. This method would not be appropriate for behavioral or ecological studies of cephalopod inking, but would be fine for prospecting for chemicals as drugs for human uses. In the following sections, I will not point out how ink is collected in every study discussed, but only in those where it is important to do so. 

### 4.2. General Composition of Ink

As described in Section 3.1, cephalopod ink is a mixture of the co-secretions from the ink sac and funnel organ. While this co-secretion has been used in some behavioral studies of squid ink [54,55], chemical analyses of the contents of the funnel organ have not been reported. Thus, what is known about the chemistry of cephalopod ink comes solely from studies of the ink sac.

Melanin has received by far the most attention, undoubtedly because it provides the distinctive black color of cephalopod ink, is a major component of ink and has been used in comparative studies of melanogenesis (see the reviews by Palumbo [57] and Prota [61]). Each ink sac of *Sepia* has ~1 g of melanin [62], and melanin constitutes ~15% of the total wet weight of ink [63]. Proteins make up another 5%–8% of the weight of *Sepia* ink [61].

### 4.3. Melanin

#### 4.3.1. What Is Melanin?

Melanin is a natural pigment that occurs in most organisms, including animals, plants, fungi and bacteria, where it has a variety of functions. It is derived from amino acids, but is not a protein. Rather, it is a complex biopolymer that typically comes in two forms, eumelanin and pheomelanin, which differ in their molecular precursors. Eumelanin is a polymer of 5,6-dihydroxyindole (DHI) and 5,6-dihodroxyindole-2-carboxylic acid (DHICA), which are derived from tyrosine. Pheomelanin is composed of the monomers, benzothiazine and benzothiazole, formed when cysteine is present. Eumelanin is dark brown in color, and pheomelanin is orange-red. Eumelanin is the form found in ink of cephalopods [57,61]. Within the cephalopods, melanin’s structure and synthesis are best characterized for cuttlefish, especially *Sepia officinalis*. Thus, most of the descriptions in this section are for melanin from *Sepia*, though some findings have been verified in other cephalopods.

*Sepia* eumelanin is a mixture of polymers that are highly cross-linked and irregular, composed of DHI and DHICA, DHI- and DHICA-derived units that are irreversibly and partially degraded, pyrrolecarboxylic acids, leucodopachrome and uncyclized DOPA units [57,64,65]. When studying such highly-complex structures, the techniques used can determine outcomes [56,57]. Using atomic force microscopy and care in isolation and purification, *Sepia* melanin has been found to exist as aggregations of particles of 100–200 μM in diameter and composed of small oligomeric units with a 5:1 DHI:DHICA ratio [56,57,66,67].

#### 4.3.2. Production of Melanin in Cephalopods

**Figure 4:**
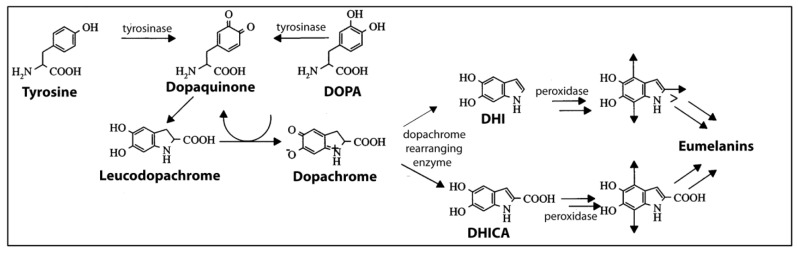
Biochemical pathways in the melanogenesis in *Sepia*. Modified with permission from Prota [61], Copyright © 2000 John Wiley & Sons, Inc. Arrows in the molecules on the right end of this figure indicate sites of polymerization. DHI, 5,6-dihydroxyindole. DHICA, 5,6-dihodroxyindole-2-carboxylic acid. DOPA, l-3,4-dihydroxyphenyl-alanine.

A summary diagram of the pathways leading to the production of eumelanin in *Sepia* ink glands is shown in Figure 4. The substrates, tyrosine and DOPA, are converted to dopaquinone by tyrosinases. Dopaquinone is unstable and non-enzymatically converts to dopachrome. Dopachrome-rearranging enzymes (DREs) then catalyze the rearrangement of dopachrome into DHI or DHICA. Then, peroxidases specific to ink glands catalyze the polymerization of DHI and DHICA monomers into eumelanin. The finding that *Sepia* melanin is composed of small oligomeric units with a 5:1 DHI:DHICA ratio does not match exactly with the catalytic properties of DREs. Palumbo [57] speculates that this apparent discrepancy may be due to the presence in the ink sac of undefined biochemical pathways, perhaps involving metals, such as copper, that can favor catalytic rearrangement of dopachrome to DHICA.

**Figure 5:**
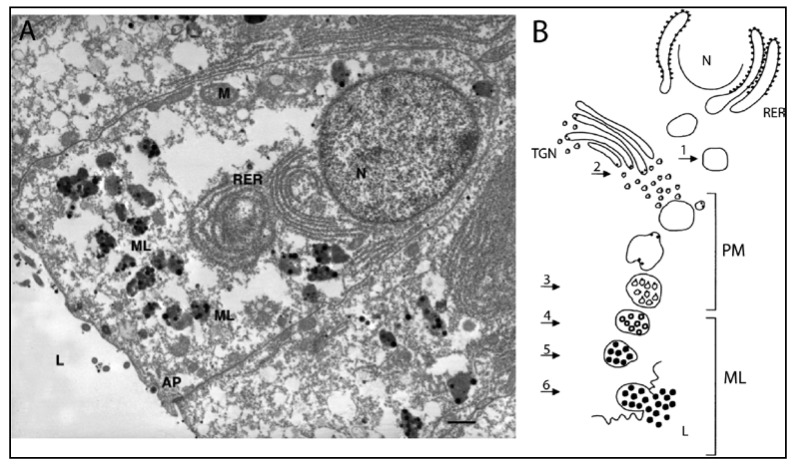
Melanocytes and melanogenesis in the ink glad of *Sepia*. (**A**) Transmission electron micrograph of a melanin-producing cell in the epithelium of an ink gland. The cell’s apical pole (AP) faces the lumen of the ink gland (9 of Figure 4C), and the nucleus (N) is near the basal pole. Melanosomes (ML) containing melanin (black dots) are located between the rough endoplasmic reticulum (RER) and the AP. Bar, 1 μM (From Palumbo [57], photo by A. Di Cosmo, used with permission); (**B**) Schematic diagram of melanin formation in a cell as in A. Steps 1–6 are described in the text. M, mitochondrion; PM, premelanosome; TGN, trans-Golgi network. Black dots in Steps 1 and 2 represent catalytic sites of melanogenic enzymes (Reproduced with permission from Palumbo [57], Copyright © 2003 John Wiley & Sons, Inc.).

These biochemical steps in melanin production have been linked to specific subcellular sites in the melanin-producing cells in the ink gland, through the work of Anna Di Cosmo, Anna Palumbo and colleagues in Naples. Their model is presented in Figure 5.

The ink gland is composed of two zones along a developmental sequence: an inner zone containing immature cells and a larger outer zone with cells that produce and secrete melanin [57,68,69,70,71]. The inner zone contains immature epithelial cells that lack pigment granules, though they do possess tyrosinase. The outer zone contains mature epithelial cells arranged in a mono-layer along the basement membrane. These are the melanin-producing cells, one of which is depicted in Figure 5A. On the cell’s basal pole is a large nucleus; on the apical pole are particulate melanosomes with different degrees of melanization, and in between is an extensive array of rough endoplasmic reticulum.

Melanosomes form in two steps, shown in Figure 5B. In Step 1, rough endoplasmic reticulum forms premelanosomes that contain active peroxidases. In Step 2, the trans-Golgi network produces vesicles containing tyrosinase and DREs. These vesicles fuse with the premelanosomes and are inverted within them, forming “coated vesicles” that present the catalytic sites of tyrosinases and DREs to the melanosome matrix (Step 3). Thus, tyrosinases and DREs are expressed in a different subcellular locus than the peroxidases involved in polymerization. Melanization occurs on the outside of the coated vesicles (Step 4), until melanin completely covers their surfaces, thus forming particulate melanosomes (Step 5). Eventually, the melanosomes fuse with the cell membrane at the apical pole, rupturing and releasing the melanosomes’ contents, including melanin, into the lumen of the ink sac (Step 6). Each melanosome makes and releases up to 30 melanin granules, each ~200 nM [56,57,66,69]. It is unknown how much melanin is secreted before the melanosomes rupture and release their contents [57].

The ink gland appears to be regulated by a signaling pathway involving glutamate, nitric oxide and cGMP, as discovered by Di Cosmo, Palumbo and colleagues [38,39,43,44,57,72]. Activation of this pathway increases tyrosinase activity and melanin production, thus likely playing an important role in the maturation and function of the ink gland.

**Figure 6:**
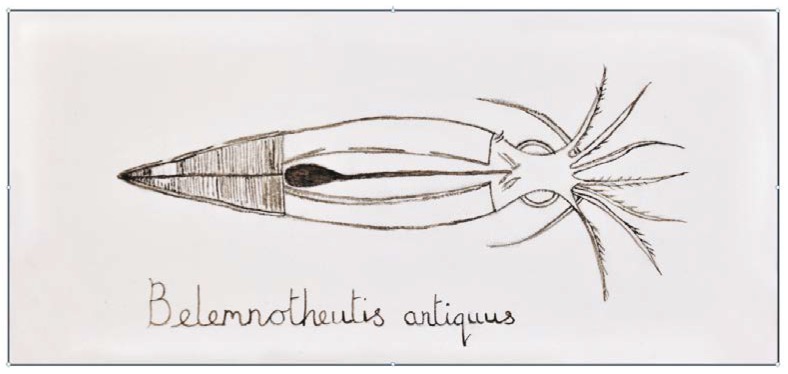
The squid, *Belemnotheutis antiquus*, based on a reconstruction from fossils acquired from a Victorian excavation in England by Glass *et al.* [73] and drawn using Jurassic-aged ink acquired from those fossilized *B. antiquus*. Reproduced by permission of the British Geological Survey © NERC. All rights reserved (CP14/029).

Melanin in extant cephalopods appears to have changed little since the Jurassic. Ink extracted from fossilized ink sacs of a Jurassic cephalopod, *Belemnotheutis antiquus*, was found to contain eumelanin with a chemical composition and structure highly similar to that of *Sepia officinalis* [73]. In fact, this 160 million year old ink was so well preserved that it was used as sepia (Section 6.2) to make a drawing of the reconstructed squid (Figure 6).

### 4.4. Melanin-Related Compounds

As described in the previous section, the melanin-producing pathway in the ink gland has a number of important chemicals, including tyrosine, dopamine and DOPA, and enzymes, such as tyrosinases, peroxidases and dopachrome-rearranging enzymes. The properties of these chemicals are described in this section.

#### 4.4.1. Tyrosinases

Prota *et al.* [62] report tyrosinase levels of 1 U per mL of ink, which is the same as 1 mg of commercial mushroom tyrosinase. This tyrosinase activity can be either tyrosine hydroxylase or DOPA oxidase, with the consumption of tyrosine (with DOPA as a co-factor, serving as a hydrogen donor) and the formation of dopachrome and a smaller amount of DOPA [62]. Tyrosinase of *Sepia* has a preference for d-isomers of tyrosine and DOPA, unlike most other tyrosinases [62]. Madaras *et al.* [53] did not find tyrosinase or other proteins in ink collected by the syringe method, but they did by the milking method.

#### 4.4.2. Dopachrome Rearranging Enzymes

A dopachrome rearranging enzyme (DRE) is present in *Sepia* ink, is associated with melanosomes, occurs as a complex with other melanogenic enzymes and catalyzes the formation of DHI and DHICA, the precursors to eumelanin [57,70,74]. DREs in *Sepia* ink preferentially yield DHI, as do some insect DREs, but unlike mammalian DREs, which preferentially yield DHICA. However, the substrate preferences for *Sepia* ink DRE and insect DREs are different.

#### 4.4.3. Peroxidases

Peroxidases are associated with melanosomes in the ink gland [57,75], where they are thought to catalyze the formation of eumelanin from DHI and DHICA [76]. A peroxidase specifically expressed in the ink gland has been cloned and sequenced [77].

#### 4.4.4. Catecholamines

The catecholamines, DOPA and dopamine, which are monoamines derived from tyrosine, are substrates of tyrosinase and are reported in the ink of several cephalopods at concentrations ranging from low nanomolar to low micromolar. Lucero *et al.* [59], using a variation on the milking method, reported that ink of the squid, *Loligo opalescens*, contains, on average, ~1 mM of DOPA and 190 μM of dopamine. Madaras *et al.* [53], using the syringe method, estimated low to mid-micromolar amounts of DOPA and dopamine in the ink of the squid, *Sepioteuthis australis*. Russo *et al.* [78] reported that expressed ink of *Sepia officinalis* contains ~1–500 nM DOPA and ~1 nM dopamine. Whether the differences in the reported levels of these compounds are due to species, the method of collecting ink or the condition of the animals is unknown. Palumbo [57] points out that while dopamine and DOPA are substrates of tyrosinase when present in cells in the ink gland, once released into the ink sac, their fate, including how they might be metabolized there, is unknown. Along these lines, 8-hydroxy-4-quinolone has been reported in the ink of the giant octopus, *Octopus dofleini martini* [79].

### 4.5. Peptidoglycans

Fucose-rich peptidoglycans have been isolated from ink of several species of squid, including *Illex argentines*, *Ommastrephes bartrami* and *Sepiella maindroni* [80,81,82,83,84,85,86,87,88,89]. These macro-molecules have largely been studied for their medicinal qualities, especially as anti-cancer agents (Section 6.1.2), rather than for their natural anti-predatory activities. Consequently, they are derived from enzymatically degraded ink and the resultant molecular fragments—polysaccharides and oligopeptides—are then purified, chemically characterized and tested using *in vitro* bioassays. The remainder of this section describes the chemistry of these molecules. Their medicinal activity will be presented in Section 6.1.

**Figure 7:**
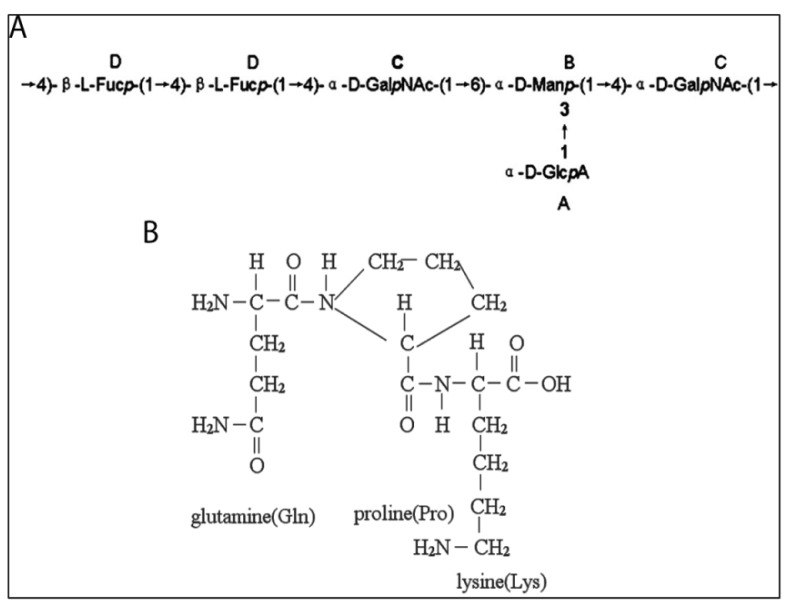
Peptidoglycans from squid ink. (**A**) Repeating unit of *Sepiella maindroni* ink polysaccharide (SIP) (Reproduced with permission from Liu *et al.* [86], Copyright © 2008 Elsevier); (**B**) *Sepia* ink oligopeptide (SIO) from *Sepia esculenta*. (Reproduced with permission from Huang *et al.* [89])

Peptidoglycans from melanin-free, papain-treated squid ink are branched glucosamine glycans having glucuronic acid-fucose (GlcA-Fuc) di-saccharide repeats in the main chain and an *N*-acetylgalactosamine (GalNAc) branch at Fuc Position 3, that is –[3GlcAβ1–4(GalNAcα1–3)Fucα1]_n_– [80–83]. Polysaccharides and oligopeptides that are constituents of these peptidoglycans have been characterized (Figure 7).

One constituent of the peptidoglycans is a polysaccharide isolated from ink of the cuttlefish, *Sepiella maindroni*, that was sequentially digested with the proteolytic enzymes, trypsin and pronase [86]. This digestion yielded a hetero-polysaccharide, called *S. maindroni* ink poly-saccharide, or SIP. SIP is composed of a repeating-unit backbone of a hexa-saccharide that is composed of glucuronic acid (labeled A in Figure 7A), mannose (labeled B in Figure 7A), *N*-acetylgalactosamine (labeled C in Figure 7A) and fucose (labeled D in Figure 7A) in a molar ratio of 1:1:2:2. This molecule has a single branch of glucuronic acid at the C-3 position of mannose and a molecular mass of 11.3 kDa [86].

Another constituent of the peptidoglycans is an oligopeptide, called *Sepia* ink oligopeptide (SIO), extracted from minced ink sacs (collected from food processing factories) by hydrolysis with trypsin after removal of melanin [83,88,89]. SIO has a tripeptide backbone consisting of *N-*Gln-Pro-Lys and a molecular mass of 343.4 Da (Figure 7B).

The bioactivity of these peptidoglycans and their derivatives as potential medicinal drugs is presented in Section 6.1.

### 4.6. Amino Acids

Cephalopod ink is high in dissolved free amino acids. Derby *et al.* [90] showed that six species of cephalopods, including two squid (*Doryteuthis* (*Loligo*) *pealeii*, *Lolliguncula brevis*), two octopuses (*Octopus vulgaris*, *Octopus bimaculoides*) and the cuttlefish, *Sepia officinalis*, have millimolar levels of total dissolved free amino acids, with levels ranging from 0.5 to 132 mM. Taurine constitutes ~50% or more of these amino acids in ink for five of the six species, and glutamate is typically one of the next highest, at three to 7%. Tyrosine levels ranged from zero to 2.9 mM, which is zero to 2% of the ink’s total dissolved free amino acids. Shirai *et al.* [91] showed that ink from three other species of squid is also rich in taurine. A potential function of these amino acids, by providing phagomimetic properties to ink in its use as a chemical defense, is described in Section 5.1.

### 4.7. Metals

Relatively high levels of metals, such as cadmium, copper, and lead, have been found in cephalopod ink. The reason for their presence is not clear, but one possibility is that they function as modulators of the activity of enzymes in the melanin synthetic pathway [92,93,94]. *Sepia* eumelanin has considerable affinity for metal ions and, as such, might serve as a trap for them [95]. This might then be related to another possible function of melanin, in excretion or detoxification [96,97].

### 4.8. Toxins

Cephalopod ink is not generally known to contain toxins. Tetrodotoxin (TTX), which is toxic to some marine animals and humans, has been found in the blue-ringed octopus, *Hapalochlaena lunulata* [98,99]. This species has TTX in its salivary glands, and a bite releases enough TTX to kill a human, especially since an anti-venom to TTX is not available. The blue-ringed octopus uses salivary TTX for capturing prey and as a defense from predators [98,99]. This species also has TTX in its ink. While the function of TTX in ink has not been experimentally tested, Williams and Caldwell [98] speculate that it may play a defensive function. The TTX is not synthesized by the octopus itself nor is it acquired in the animal’s diet; rather, it is produced by symbiotic bacteria.

Dietary sources of toxins in cephalopod ink have not been reported. In one case, where dietary toxins were investigated in cephalopods, domoic acid, which is a food web-transferred algal toxin in some organisms, was found in a few organs of *Octopus vulgaris*, but not in the ink sac [100]. Nonetheless, dietary-derived toxins remain theoretically possible for cephalopods.

## 5. Ink as an Anti-Predator Defense

### 5.1. Interspecific Effects: Ink as a Direct Deterrent of Predators

Inking is a defense coupled to jetting and changes in body coloration, such as in the blanch-ink-jet maneuver [2,101]. Ejected ink can take several forms, including diffuse clouds of black ink containing relatively little mucus, darkened blobs with relatively more mucus and called pseudomorphs because they generally resemble the form of a cephalopod, and mucousy secretions called ropes because they are longer and thinner than pseudomorphs [6,8,34]. Actually, the diversity of types might be higher, or there may not even be types, but rather a continuum of different forms. This is supported by Bush and Robison’s [6] description of six types of ink released from deep-sea squid, which they classify as pseudomorphs, pseudomorph series, ink ropes, clouds/smokescreens, diffuse puffs and mantle fills.

These different forms of ink might defend cephalopods against predators in a variety of ways. Most obvious are the mechanisms operating in the visual channel of predators. In this way, ink clouds could serve as smoke-screens behind which inking cephalopods can hide or jet escape. Pseudomorphs might mimic the form of the inking cephalopod and, by attracting the attention of the predator, buy the cephalopod time to escape [102]. Ink ropes, by virtue of resembling elongate siphonophores, might serve as visual mimics of these stinging animals and allow deep-sea squid to escape [6]. Bush and Robison [6] also note that deep-sea *Heteroteuthis* squid have ink sacs and produce an ink containing mucus and luminescent bacteria from their light organs, thus creating luminescent clouds, which the squid might use as a visual defense to either conceal themselves or confuse predators. 

Observational studies in the field and laboratory amply support the idea that ink defends cephalopods from predators. For example, Staudinger *et al.* [103] show in laboratory experiments that inking by *Doryteuthis pealeii* is correlated with changes in the attack behavior of predatory fishes, such as increases in startle behavior, abandonment of attacks by bluefish and misdirected attacks by flounder. Experimental studies are rarer and limited to laboratory simulations of predator-prey interactions, but they support a defensive role of ink by showing that a cloud of ink released between a piece of food and an approaching fish will slow the fish’s attack [55,104].

The possibility that ink may act as a chemical defense against predators has been suggested from anecdotal observations, such as octopuses squirting ink at snails or crabs approaching their eggs [105] and cuttlefish placing ink in their egg capsules [4,106]. Boyle and Rodhouse [4] concluded that “cephalopods do not appear to have evolved any chemical defenses.” This conclusion is based on their assertion that “generally the cephalopods produce no toxins to provide a disincentive to consumption by oceanic higher predators.” While this statement is generally true (see an exception about TTX in blue-ringed octopus, in Section 4.8), many chemical defenses that organisms produce, including cephalopods, are not toxins. Rather, these chemicals can defend by being attractants or repellents, which might be used in mimicry, camouflage and other mechanisms, as shown for a variety of animals [107,108,109]. Indeed, some of these mechanisms have been suggested for cephalopod ink. For example, ink has been proposed as an aversive deterrent or as a disruptor of predators’ chemical senses [62,110,111,112,113,114]. Ink has also been proposed as a candidate phagomimetic defense, due to its high levels of amino acids (Section 4.6), which are strong phagostimulants of marine predators, including fish. Accordingly, the phagomimetic properties of ink would cause predators to attend to the ink, thus giving cephalopods more time to escape, as has been shown for sea hares (Figure 2D) [115].

Recent experimental tests have supported the idea that cephalopods use ink as an anti-predatory chemical defense. Ink from two species of squid, the Caribbean reef squid, *Sepioteuthis sepioidea*, and the longfin inshore squid, *Doryteuthis pealeii*, is unpalatable to predatory fish and, as such, might provide a defense during attacks [55,104]. The molecular identities of these deterrents are unknown. The fraction of ink containing melanin granules has most of the deterrent activity, suggesting that compounds adhering to these granules or melanin itself might be unpalatable. Prota *et al.* [62] speculate that feeding deterrents in *Sepia* ink might be quinones, since they are produced by tyrosinase’s catalysis of the oxidation of phenols. This hypothesis is supported by the observations that ink of the giant octopus, *Octopus dofleini martini*, contains 8-hydroxy-4-quinolone [79] and that quinones and related compounds are often used as chemical defenses by animals [116]. Tests of cephalopod ink as a phagomimetic defense have yielded mixed results. Ink from two species of squid was not found to be phagostimulants for predatory fish [55,104]. However, ink from octopus ink was found to be attractive to moray eels, evoking searching and attack behaviors, much as did food-related chemicals [117]. To date, there are no reports of experimental tests of cephalopod ink as an inactivator or disruptor of the chemical senses.

**Figure 8:**
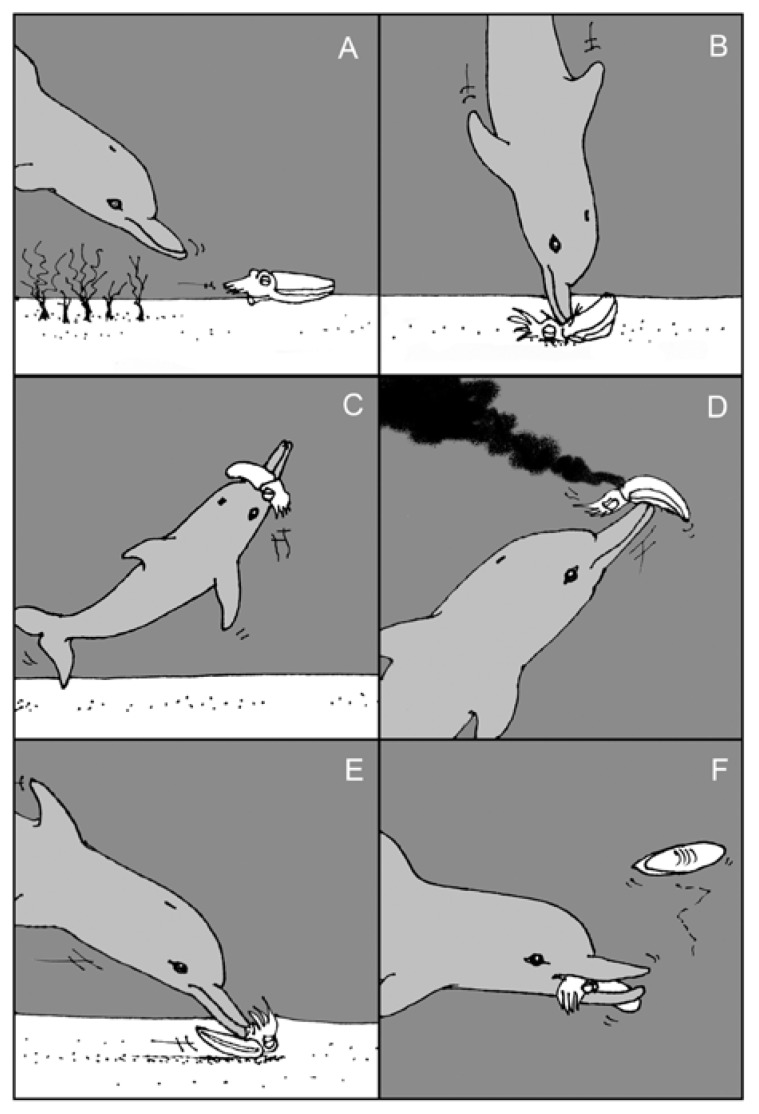
Handling of giant cuttlefish *Sepia apama* by Indo-Pacific bottlenose dolphin *Tursiops aduncus*. In this sequence, the prey is: (**A**) chased from the algal-covered substrate into the open, (**B**) pinned to the substrate and killed, (**C**) carried towards the surface, (**D**) beaten against the substrate with the dolphin’s snout to release ink, (**E**) returned to substrate, inverted and forced along the sand to remove skin and cuttlebone, and (**F**) consumed. (Reproduced with permission from Finn *et al.* [118])

Of course, cephalopods are sought by many predators, and the cephalopods’ defenses, including inking, are not always sufficient to prevent successful attacks. Dolphins can cleverly elude the chemical defenses of cuttlefish by de-inking them after capture and before consumption, as shown in Figure 8 [118]. This behavior is reminiscent of that of rodents when attacking insects that produce noxious chemicals, wherein the rodents stick the chemical-secreting end of the insect into the ground, to avoid that danger, and eat starting at the other end of the insect [119,120].

### 5.2. Intraspecific Effects: Ink as an Alarm Cue for Conspecifics

A second type of anti-predatory chemical defense in the ink of cephalopods functions indirectly—not by acting on the predators themselves, but rather are alarm cues for conspecifics. Ink can cause jetting behavior in *Loligo opalescens* [59,121] and cryptic (camouflage), deimatic (threatening or startling) and protean (unpredictable) behaviors in *Sepioteuthis sepioidea* [54]. DOPA or dopamine at biologically-relevant concentrations is sufficient to evoke jetting in *L. opalescens* [59,121]. Melanin may play a role in the effects of these catecholamines. Melanin adsorbs dopamine, allowing melanin to be possibly a carrier of dopamine in ink [58]. Moreover, melanin is an antioxidant/reductive agent, so it might stabilize DOPA and dopamine by preventing their oxidation [58]. In addition, ink may contain other antioxidants [59].

### 5.3. Evolution of Melanin as a Defense

How might melanin have evolved as a component of the ink defensive secretion of cephalopods? Melanin is a scavenger of free radicals, and as such, it can play a photoprotective role, including in vision [122]. Therefore, melanin may have initially functioned as a photoprotectant in the eyes, skin or other tissues of ancestral cephalopods. Melanin in the skin of cephalopods may have then taken on a secondary function of camouflage, both passive and active, for which cephalopods are renowned [2,4,101]. The ink sac may have had an early function in excretion (in addition to the traditional excretory organs, the nephridia), playing a role in the excretion of melanin. Subsequently, the ink sac may have evolved its specific defensive function, with melanin taking on a new role as an anti-predatory defense. Melanin’s role as a visual cue in defense is clear, even if its role as a chemical defense is still uncertain, either as a defensive compound itself or as a carrier of other, bioactive compounds (Section 5.1). Interestingly, pigmented molecules, such as melanin, are rarely reported as being deterrents, though there are exceptions, as reviewed in [123].

## 6. Human Applications of Cephalopod Ink

Cephalopod ink has been used by humans for many practical and commercial purposes over the millennia, especially in medicine, cuisine and art, but in even broader applications, as described in this section.

### 6.1. Drugs

Cephalopod ink has a long history of being used to promote human health. Nair *et al.* [124] provide a review of the medicinal effects of not only ink, but other tissues of cephalopods. Many health benefits have been ascribed to cephalopod ink as a traditional medicine, both in Western culture (ancient Greece and Rome) and Eastern culture (China) [124,125]. More recently, cephalopod ink has been used in an attempt to develop new drugs, through the search for new natural compounds with beneficial health effects. This is an especially active field in Asia, where cephalopods are a major fishery catch, for which ink sacs are a bi-product and where homeopathic medicine has deep roots. It should be noted that the ink used in drug discovery is not always fresh, often being taken from dead animals, sometimes from preserved animals. Homogenized ink sacs are often used as the source material, and this is then digested or chemically processed. Therefore, caution must be used in assuming that compounds identified through this process of drug discovery might also be present in naturally released ink and used by cephalopods in their natural environment. Furthermore, since such drug discovery involves intellectual property with commercial applications, identified bioactive compounds may not be reported. Consequently, published work in this field tends toward phenomenology in which effects are identified, but underlying molecules or mechanisms are not. The following section reviews some of these effects.

#### 6.1.1. Antimicrobial Properties

Cephalopod ink has antimicrobial properties against a diversity of organisms, including human pathogens [126,127,128,129,130,131]. Antimicrobial activity is found in various extracts of ink, including aqueous [132] and organic solvents [133,134], and in the melanin fraction [135,136]. The molecular identities of these antimicrobials, if known, are not reported in the literature.

#### 6.1.2. Potential Anticancer Properties

Cephalopod ink has potential as an anticancer agent, based on *in vitro* studies of various cell types and cell lines [60,78,137]. The effect is often through the induction of apoptosis and is often associated with different chemicals in ink. The specificity of the effects on cancerous cells is not often explored in studies in this field.

Tyrosinase (Section 4.4.1) extracted from the melanin-free fraction of ink is toxic to transformed human cell lines [78]. This effect is mediated by purified tyrosinase with no added substrate, so the substrate is probably provided by the human cells themselves. Russo *et al.* [78] speculate that this apoptotic effect is due to the production of dopaquinone, which is known to interact with nucleophiles to produce protein-bound DOPA through a 5-*S*-cysteinyldopa residue, which, in turn, can oxidatively damage cellular molecules.

Peptidoglycans from squid and cuttlefish ink (Section 4.5) can have anti-tumor effects [60,81,84,123], which is perhaps not surprising, since in eukaryotes in general, peptidoglycans can affect cell division [138]. Mechanisms underlying the effects of cephalopod peptidoglycans may include fragmentation of DNA [139] and apoptosis [123], perhaps resulting in the inhibition of embryonic development [139].

*Sepiella maindroni* ink polysaccharide (SIP), derived by enzymatic digestion of the peptidoglycans (Section 4.5), when treated with chlorosulfonic acid, yields a sulfated SIP, called SIP-SII, having a sulfate content of ~35% [85,86,87]. SIP-SII has anti-cancer activity, which may result from several of its properties: (1) suppression of the invasion and migration of carcinoma cells via inhibition of matrix metalloproteinase-2 [85]; (2) suppression of melanoma metastasis via inhibition of tumor adhesion mediated by intercellular adhesion molecule 1; and (3) inhibition of angiogenesis mediated by basic fibroblast growth factor [87].

*Sepia* ink oligopeptide (SIO), extracted from enzymatically digested ink sacs (Section 4.5) [83,88,89], also has anti-cancer properties. Its mode of action in prostate cancer cells is by induction of apoptosis via activation of caspase-3 and elevation of the ratio of Bax/Bcl-2 [88,89].

#### 6.1.3. Hematopoietic Effects

Cuttlefish ink may enhance immune responses by affecting hematopoiesis. For example, it promotes the proliferation and differentiation of granulocyte-monocyte progenitor cells [140].

#### 6.1.4. Anti-Hypertensive Actions

An angiotensin-converting enzyme purified from squid ink causes dilation of blood vessels, resulting in lower blood pressure. This represents a potential treatment of hypertension [141]. The bioactivity can be traced to peptide derivative of ~294 Da [141].

#### 6.1.5. Anti-Retroviral Activity

Ink from *Loligo duvauceli* and *Sepiella inermis* has been reported to have an anti-retroviral activity [142].

#### 6.1.6. Potential Anti-Ulcerogenic Actions

Ink from squid and octopus inhibits gastric secretion of rats and, thus, has potential in the development of anti-ulcerogenic drugs [143,144,145,146]. The active fraction contains an unidentified low molecular weight melanoprotein that might be responsible for the activity, by enhancing the glycoprotein activity in the gastric mucosa.

#### 6.1.7. Anti-Inflammatory Activity

Mimura *et al.* [146] reported anti-inflammatory activity for the same fraction of squid ink that inhibits gastric secretion, as described in Section 6.1.6. Fahmy and Soliman [137] reported anti-inflammatory effects of *Sepia* ink.

#### 6.1.8. Anti-Oxidant Activity

Cephalopod ink has anti-oxidant activity [137,140,147,148]. Activity resides in both the melanin and melanin-free fractions of ink [147,149]. This anti-oxidant activity may be related to some of the other effects reported in Section 6.1, such as anti-cancer effects, as well as its photoprotective effects [122,135].

### 6.2. Sepia Ink as a Pigment in Writing, Art and Cosmetics

**Figure 9:**
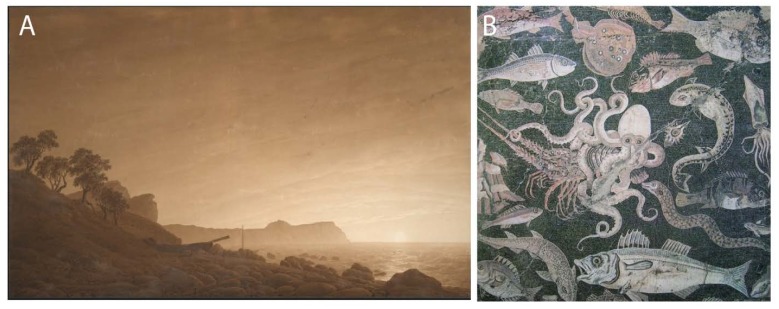
Cephalopods in art. (**A**) Drawing in sepia by Caspar David Friedrich, “View of Arkona with Rising Moon” (*ca.* 1805) in the Albertina, Vienna); (**B**) Mural from Pompeii (*ca.* 45–79 AD) in the Museo Archeologico Nazionale di Napoli, with Mediterranean sea animals, including octopus, attacking a spiny lobster.

Due to its color and permanence, sepia—the name used for the black ink from *Sepia*—was extensively used from Greco-Roman times through the 19th century as an ink and pigment used in writing, drawing and painting (Figure 9A). It can be diluted to yield various shades. Sepia is available and used even today, though modern dyes and pigments with similar hues and other advantages have largely replaced sepia. A modern drawing using ink from a fossilized squid is shown in Figure 6. Cephalopods over the ages have been not only a source of artistic materials, but of artistic inspiration. One example, a painting of marine life, including an octopus attacking a spiny lobster, is shown in Figure 9B.

With the goal of developing eye cosmetics that are both effective and that support “sustainable development with respect to the environment and health,” mascara, eyeshadow and other eye cosmetic products using sepia have been and are being developed [150,151,152].

### 6.3. Cephalopod Ink as Food

Cephalopod ink has been used in various ways in the food industry. Most common is its use as a food flavoring, used worldwide. Most commercially-sold squid ink is actually cuttlefish ink, because of its superior flavor [153]. *Arroz negro* (black rice), *txipirones en su tinta* (baby squid in ink sauce), *ikasumi jiru* (ink soup with pork and squid) and Cavianne (an imitation caviar) are some of the dishes and foods that use cephalopod ink. Processed ink is used as a food coloring [154]. Due to its antimicrobial properties (see Section 6.1.1), cuttlefish ink is also used to cure and, thus, extend the shelf life of cuttlefish meat [126,128,155].

## 7. Future Directions

Inking is a defining behavior of cephalopods that has attracted the interest of humans for millennia. This review has summarized what (little) is known about inking behavior and ink. This section offers some directions that future studies of cephalopod ink and inking might take.

In the realm of neuroecology [156], what is known about the role of ink in defense against predation is almost entirely from observational studies. Experimental studies are needed to identify molecules and mechanisms underlying ink behavior. In terms of behavior, experimental studies, in which the release of ink from behaving cephalopods is manipulated, towards identifying ink’s roles as inter- or intra-specific defensive cues, as has been done for other inking mollusks [115,157], have not been reported. Through which sensory channels—visual, chemical, mechanical—does ink affect predators and how? Other than the extensive work on melanin and its synthesis, very little is known about the chemistry of ink, so this is a priority, particularly from a functional perspective. The combined experimental approaches of bioassay-guided fractionation and biomarker targeting will likely be necessary to identify the bioactive molecules in ink, whose chemical composition is complex. Laboratory studies need to be informed by, grounded in and extended to field observations and experiments. Is there a functional basis to the various forms of released ink—clouds, ropes, pseudomorphs, and so on? Might these different forms defend individuals in different ways against diversity predators? Almost nothing is known about the funnel organ, which produces the mucus component of ink. What chemicals are in the funnel organ’s mucousy secretion? Likely it is to be much more than just mucus, similar to the opaline gland of sea hares, which, together with the ink gland, produces a rich array of bioactive molecules (Figure 2D) [158]. The innervation of the ink sac and funnel organ has received very little attention, and it is not even clear at present if the two organs are controlled by the same or different neural centers. Whether there are differences in signaling pathways (neurotransmitters, second messengers, modulators) for the two organs is also unknown. Knowing the independence of the two pathways is essential in determining the mechanisms underlying the production of the different forms of released ink.

Experimental approaches to gaining a molecular and biochemical understanding of inking are now available. So far, little has been done along these lines. Several individual genes from the ink sac involved in melanin synthesis have been cloned, for comparative studies of enzymes. These include peroxidase [77], tyrosinase [159] and nitric oxide synthase [160]. One transcriptome of the ink sac has been generated, which identified another gene involved in melanogenesis—arginine kinase—as well as other genes that are involved generally in metabolism and, therefore, that might play a role in the production, mobilization and release of ink [161]. Much more can and should be done with molecular tools, such as transcriptomics, RNA inhibition and optogenetics, to understand ink’s form and function.

Ink has a diversity of benefits to offer us through industrial and medical applications. Two major potential benefits are identifying antimicrobials to treat products used in food, cosmetics and healthcare and developing drugs for use as antimicrobials, anti-cancer, anti-oxidants and more. Our current knowledge of such uses is largely phenomenological, and the chemicals in ink responsible for the effects and the underlying cellular or molecular mechanisms have not been identified. It will be most useful to find anti-microbial drugs that act through novel mechanisms, given the prevalence of drug-resistant micro-organisms. Natural products have often offered new avenues for drug development [162,163], and given the diversity of medicinal effects of whole cephalopod ink, it offers promise for identifying new, prospective drugs.

## 8. Conclusions

This review has summarized what is known about cephalopod ink, particularly related to its production, chemical composition, use by cephalopods in predator-prey interactions, and human applications in drug discovery and development. It is evident from this review that cephalopod ink has been appreciated by humans for centuries, yet so little is known about it. It is hoped that this review encourages a deeper look at cephalopod ink, both as a mechanism of defense for these beautiful animals and as a source of drugs for human consumption.
